# A topology-dynamics-based control strategy for multi-dimensional complex networked dynamical systems

**DOI:** 10.1038/s41598-019-56259-4

**Published:** 2019-12-27

**Authors:** Mohammadreza Bahadorian, Hamidreza Alimohammadi, Tahereh Mozaffari, Mohammad Reza Rahimi Tabar, Joachim Peinke, Klaus Lehnertz

**Affiliations:** 1 Department of Physics, Sharif University of Technology, Tehran, 11155-9161 Iran; 2 Institute of Physics and ForWind, Carl von Ossietzky University, 26111 Oldenburg, Germany; 3 Department of Epileptology, University of Bonn, Venusberg Campus 1, 53127 Bonn, Germany; 4 Helmholtz Institute for Radiation and Nuclear Physics, University of Bonn, Nussallee 14–16, 53115 Bonn, Germany; 5 Interdisciplinary Center for Complex Systems, University of Bonn, Brühler Straße 7, 53119 Bonn, Germany

**Keywords:** Physics, Complex networks

## Abstract

Complex systems are omnipresent and play a vital role in in our every-day lives. Adverse behavior of such systems has generated considerable interest in being able to control complex systems modeled as networks. Here, we propose a topology-dynamics-based approach for controlling complex systems modeled as networks of coupled multi-dimensional dynamical entities. For given dynamics and topology, we introduce an efficient scheme to identify in polynomial time a finite set of driver nodes, which – when endowed with the control function – steer the network to the desired behavior. We demonstrate the high suitability of our approach by controlling various networked multi-dimensional dynamics, coupled onto different topologies.

## Introduction

Emergent behaviour of complex systems can be either desired (e.g. learning or intelligence) or undesired (e.g. hurricanes or cascading failures)^1,2^. Generally, one aims to retain complex systems closer to the desired state, and control of such systems is important in order to steer the system’s dynamics to that state (assuming that it exists)^3–5^. Contemporary control strategies can be divided into four major categories, depending on the control objective and the type of system dynamics they are suited for.

(i) *Linear systems or linearized dynamics control* aims to fully control linear systems or general non-linear systems near a nominal trajectory or a fixed point. Based on this objective, proposed strategies are based on Kalman rank condition^6^, structural controllability^7–9^, structural adaptation and contraction^10,11^, exact controllability^12^, path reachability^13,14^, and on spectral properties of the Jacobian matrix^15^.
(ii) *Non-linear systems control* initially pursued the ambitious full controllability of general non-linear systems^16–19^ but was proven to be NP-hard for some cases^20^, which led to weaker notions such as local accessibility/controllability^20^.
(iii) *Final-state-oriented control* does not actually intend to fully control the state of the system, but aims to steer the system towards a desired long-term behavior. Control through adjustments of the initial condition of the dynamics (placing the system in a new basin of attraction)^21^, control through change of parameters and exploiting network regulatory attributes^22^ and controlling chaos^23^ are known works conducted in this category. Another strategy developed recently in this category, based on the concepts of “feedback vertex set” and “determining nodes”, is the so called “feedback vertex control”^24,25^ which is almost independent of the underlying dynamics of the network and mostly depends on the wiring of the system and knowledge of the target invariant sets.
(iv) *Collective behavior control* intends to control the collective complex behavior of a system. Some well-known strategies in this category are based on the master stability formalism^26,27^ and on pinning control^28,29^, motivated by publications on chaos control^30^, and nonlinear distributed control protocols^31^ aim at identifying the required pinning term to be added to the dynamics of all nodes.

Most of these strategies solely depend on the system’s network structure and have no direct connection to the underlying dynamics, and it only suffices for the dynamics to be describable by homogeneous dynamical equations.

The complexity of state-of-the-art strategies^32^ for controlling linear time-invariant dynamics of networks with *N* nodes, and if one uses the Kalman rank condition, is 2^*N*^ times the complexity of evaluating the rank of the Kalman controllability matrix, which stems from the fact that there are 2^*N*^ − 1 possible combinations for selecting driver nodes. For strategies based on structural controllability, the minimum number of driver nodes *N*_*D*_, needed to fully control a directed network, is determined by the maximum matching in the network, where the unmatched nodes are exactly the driver nodes^8^. This allows one to find the driver nodes with the Hopcroft-Karp algorithm^33^, which has complexity ${\mathscr{O}}(\sqrt{N}L)$ where *L* denotes the number of links. Strategies based on exact controllability can be applied to arbitrary network structures and link weights without any limitations, and with the Popov-Belevitch-Hautus rank condition^34^ its complexity is ${\mathscr{O}}({N}^{2}\,{\mathrm{ln}}^{2}(N))$. Another strategy, which also solely takes into account the structure of the underlying network, is based on the minimum dominating set^35,36^ and has complexity ${\mathscr{O}}{\mathrm{(1.5137}}^{N})$^37^.

Here, we propose a control approach and a driver-node-identification scheme – based on a transformation of state variables of multi-dimensional dynamics – that makes use of both the equations of motion of the subsystems and topological properties of the underlying network. This enables us to determine a suitable control function. When endowed with this control function, the driver nodes steer the network to the desired state. Our approach belongs to the category (i) but our driver-node-identification scheme can also be combined with control strategies from category (iv) (Supplementary Information).

## Results

We illustrate our approach – based on pinning – to control a complex dynamical network towards a desired state. Our approach relies on the linearization (as well as transformation of state variables) of any given dynamics in the vicinity of a desired final state and on meeting the necessary criteria to linearly stable state. In addition, we present an algorithm that allows one to determine – for the given dynamics and topology – a small set of the required driver nodes. Our approach is established upon few assumptions that will be stated in what follows and they are set to be default in the whole paper, unless explicitly stated. First, we assume that the desired state is a fixed point of the system. We note though that one can employ our approach also in cases where the desired state is a nominal trajectory by combining it with the master stability function (MSF) formalism^26,27^ (Supplementary Information). Second, we assume full observability of the system, i.e., the state of the whole system is exactly known at any time *t*. Third, we assume accessibility of the system, i.e., each and any subsystem can be pinned by the specific control function.

### Control strategy

We present the basic ideas to determine the control function in the Methods section. Here, we illustrate our approach by controlling a network of second-order Kuramoto oscillators. The *N* phase oscillators *θ*_*i*_ (*i* ∈ {1, …, *N*}), placed on a network, evolve according to^38^

1 $${\ddot{\theta }}_{i}={\omega }_{i}-{\alpha }_{i}{\dot{\theta }}_{i}+\mathop{\sum }\limits_{j\mathrm{=1}}^{N}\,{\lambda }_{ij}\,\sin ({\theta }_{j}-{\theta }_{i}),$$

where *ω*_*i*_ denotes the unique natural frequency (deviation from a nominal frequency) of the *i*th oscillator and *α*_*i*_ is the damping constant (we fix *α*_*i*_ = 2 for all oscillators and consider *ω*_*i*_ to be uncorrelated random numbers, Gaussian distributed, with zero mean and fixed standard deviation). Also the non-zero coupling strengths *λ*_*ij*_ are independent, normally distributed, random variables with a mean in the interval [0.1; 1.5] and a standard deviation amounting to one fifth of the mean. We have chosen the values of parameters such that they allow the system to attain both synchronised and asynchronous states with different initial conditions.

**Figure 1:**
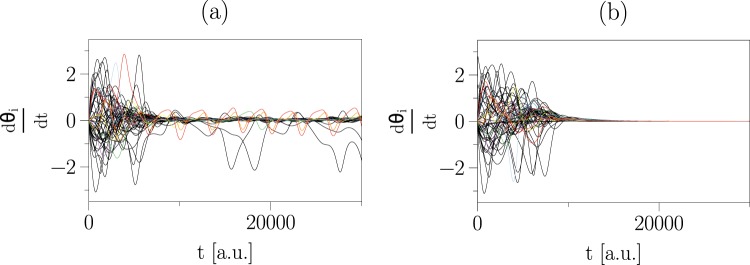
Control of networked second-order Kuramoto oscillators. (**a**) frequency dynamics of second-order Kuramoto oscillators (*N* = 120) coupled onto an Erdős-Rényi network (wiring probability *p* = 0.03) and starting from some random initial condition from which the system does not achieve the fully synchronised state. (**b**) frequency dynamics of the controlled system with *N*_*D*_ = 10 driver nodes starting from the same initial condition. The controlled system attains the synchronised state after about 20000 integration time steps (4th-order Runge-Kutta method with step size *dt* = 10^−3^).

By integrating Eq. (1) for a given network topology and initial condition, we derive the temporal evolution of variables *θ*_*i*_(*t*) and ${\nu }_{i}(t)={\dot{\theta }}_{i}(t)$, which fully determine the system’s dynamics. If the phase *θ*_*i*_(*t*) of oscillator *i* approaches the phase of the mean field (i.e., $\psi =arctan(\Im ({\sum }_{j}{e}^{i{\theta }_{j}})\Re ({\sum }_{j}{e}^{i{\theta }_{j}}))$, apart from a constant shift), it is locked to the mean field, and if this happens to all oscillators, the system is synchronised. In the left part of Fig. 1, we show an exemplary frequency dynamics of a network of non-identical second-order Kuramoto oscillators that, starting from some random initial condition, does not achieve the synchronised state. We note that for other initial conditions and network configurations the system may attain the synchronised state.

In the following, we refer to the synchronised and the incoherent state as the desired and the undesired state, respectively. Our approach consists of identifying the oscillators (or nodes) that cause positive eigenvalues of the network’s Jacobian matrix of transformed state variables (see below, i.e. Eqs. 11 and 12), and controlling them will lead to a linearly stable desired state (Methods). To do so, we inspect the spectrum of eigenvalues around the desired state. If all eigenvalues have a negative real part, the system is linearly stable around that state. We verify the vanishing of the positive real part of eigenvalues by means of the Gershgorin circle theorem^15,39^ (Methods). In order to guarantee that all components of the dynamical system will approach the desired state (i.e., all eigenvalues will lie in the negative half-plane of the complex plane), we move all Gershgorin disks to the left half-plane of the complex plane by adding a pinning term (in the general form of a feedback-control term) such as $-{\beta }_{i}({x}_{i}-{x}_{i}^{\ast })$, where *β*_*i*_ is the pinning strength and *x*_*i*_ the state variable of node *i*. Here ${x}_{i}^{\ast }$ denotes the *i*-th component of the fixed point. We note that, depending on the definition of state variables, the pinning term might find some nontrivial form as shown in two examples in the reminder of this paper.

Now, if one defines ${\dot{\theta }}_{i}={\nu }_{i}$ and ${\dot{\nu }}_{i}$ as two coupled dynamical equations, the first *N* disks (out of the 2*N* Gershgorin disks for the corresponding Jacobian matrix evaluated at the fixed point – which are associated with $\partial {\dot{\theta }}_{i}$ (here ∂ refers to partial differentiations of $\dot{\theta }$ with respect to *ν* or *θ*) – will be centered at the origin and will have unit radius each (Supplementary Information). Eigenvalues of the Jacobian matrix, which lie on the union of all Gershgorin disks, thus may possess a positive real part. This can be avoided by a change of variables by the canonical transformations ${\tilde{\theta }}_{i}={\theta }_{i}$ and ${\tilde{\nu }}_{i}={\nu }_{i}+\kappa {\theta }_{i}$, with *κ* = 1, and by rewriting the set of Eq. (1). The new governing equations then read:

2 $$\begin{array}{rcl}{\dot{\tilde{\theta }}}_{i} & = & {\tilde{\nu }}_{i}-{\tilde{\theta }}_{i}\\ {\dot{\tilde{\nu }}}_{i} & = & {\omega }_{i}+\mathrm{(1}-\alpha ){\tilde{\nu }}_{i}-\mathrm{(1}-\alpha ){\tilde{\theta }}_{i}+\mathop{\sum }\limits_{j=1}^{N}\,{\lambda }_{ij}\,\sin \,({\tilde{\theta }}_{j}-{\tilde{\theta }}_{i}\mathrm{)}.\end{array}$$

The Gershgorin disks related to $\partial {\dot{\tilde{\nu }}}_{i}$ of the corresponding Jacobian (Supplementary Information) have the following centers *C*_*i*_ and corresponding radii *R*_*i*_:

3 $$\begin{array}{rcl}{C}_{i} & = & 1-\alpha \\ {R}_{i} & = & |\alpha -1-\mathop{\sum }\limits_{k=1}^{N}\,{\lambda }_{i-N,k}\,\cos \,({\theta }_{k}^{\ast }-{\theta }_{i-N}^{\ast })|+\mathop{\sum }\limits_{j=1}^{N}\,{\lambda }_{i-N,j}|\cos \,({\theta }_{j}^{\ast }-{\theta }_{i-N}^{\ast })|\end{array}$$

for *i* = *N* + 1, *N* + 2, …, 2*N*. Nodes for which the value of *C*_*i*_ + *R*_*i*_ is positive are “problematic” since their dynamics can induce a positive real part of eigenvalues. It can be shown (Methods) that adding a term of the form $-{\beta }_{i}({\tilde{\nu }}_{i}-{\tilde{\nu }}_{i}^{\ast })$ to the r.h.s. of Eq. (2) for ${\dot{\tilde{\nu }}}_{i}$ or, equivalently, a term of the form $-{\beta }_{i}({\nu }_{i}-{\nu }_{i}^{\ast })-{\beta }_{i}({\theta }_{i}-{\theta }_{i}^{\ast })$ to the equation for ${\dot{\nu }}_{i}$, enables us to move the eigenvalues of the Jacobian to the left half-plane of the complex plane. This possible control strategy can be interpreted as pinning the variables to the values of their associated components of the desired state, which assures the stability of such a state if the pinning strength *β*_*i*_ for node *i* satisfies the following condition:

4 $${\beta }_{i}\ge 1-\alpha +|\alpha -1-\sum _{k}\,{\lambda }_{ik}\,\cos \,({\theta }_{k}^{\ast }-{\theta }_{i}^{\ast })|+\sum _{j}\,{\lambda }_{ij}|\cos \,({\theta }_{j}^{\ast }-{\theta }_{i}^{\ast })|\mathrm{}.$$

Accordingly, these problematic nodes or “driver nodes” need to be pinned.

### Identification of driver nodes

The Gershgorin circle theorem provides us with a sufficient but not a necessary condition for controlling a network of oscillators. Applying the obtained control function to the set of Eq. (2) and using condition (4) can thus result in an abundant number of driver nodes. We therefore aim at identifying a finite and smaller number of driver nodes (set of size *N*_*D*_ ≪ *N*) that mainly affect the network’s dynamics. To do so, we propose the following scheme:

- Step 1: add the control term with strength *β*_1_ satisfying condition (4) to node/oscillator 1, calculate the maximum real part of the Jacobian’s eigenvalues, and save its value as *e*_1_.
- Step 2: repetition of step 1 separately for each of the remaining *N* − 1 nodes leads to the set of values {*e*_1_, *e*_2_, …, *e*_*N*_}.

- Step 3: identify the minimum value in this set. The corresponding node will be a driver node and its control term will be fixed.
- Step 4: if *min*(*e*_*i*_) ≥ 0, repeat steps 1–3 and identify other driver nodes.

Note that the linearization of the dynamics around a fixed-point may lead to a small error for finite deviations from the fixed-point. This error can be avoided by introducing a small negative upper bound for *min*(*e*_*i*_). For the analysis presented below, we use the condition *min*(*e*_*i*_) ≥ −0.2.

The computational complexity of our driver-node-identification scheme scales with network size as *N*^*δ*^ × *m*^2.4^, where *δ* < 4.4 and *m* is the dimensionality of the state variable of a given node (e.g., *m* = 2 for the second-order Kuramoto oscillators). Applying our control strategy to the system presented in the upper part of Fig. 1, we find that only *N*_*D*_ = 10 driver nodes (out of total 120 nodes) are required to steer the system dynamics from the undesired state (asynchronous) to the desired one (synchronised, cf. right part of Fig. 1).

### Impact of coupling topology

To demonstrate extendability of our observations beyond exemplary dynamics, we now investigate how the important aspects of our control strategy – namely the required number of driver nodes *N*_*D*_ and its fraction *n*_*D*_ = *N*_*D*_/*N* (pinning density) as well as the pinning strengths *β* – depend on properties of the coupling topology. We here consider Erdős-Rényi (ER) networks^40^ (wiring probability *p* ∈ {0.03, …, 0.8}) and scale-free (SF) networks (Barabási–Albert model^41^ with an average number of neighbors $\simeq 4$), both for a range of the number of nodes (*N*∈{50, …, 290}). We generate 10 realisations for each network of coupled second-order Kuramoto oscillators.

**Figure 2:**
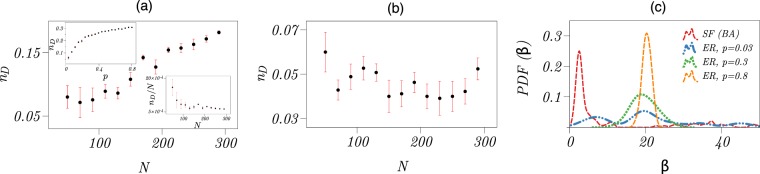
Impact of network characteristics on pinning density and strength for networked second-order Kuramoto oscillators. Dependence of pinning density *n*_*D*_ (fraction of driver nodes) on network size *N* for (**a**) Erdős-Rényi (ER) and (**b**) scale-free (SF) oscillator networks. For Erdős-Rényi networks, dependence of *n*_*D*_ on the wiring probability *p* for a given size of *N* = 120 is shown in upper inset. In the lower inset, we show the dependence of *n*_*D*_/*N* on *N* for *p* = 0.03, which saturates to a constant for large *N*. Means and error bars are from 10 realisations of each network. (**c**) Probability distribution functions (PDF, estimated using a Gaussian kernel) of pinning strengths *β* required to control SF and ER oscillator networks (with different wiring probabilities *p*). For each of the 30 network realisations, we chose the minimum pinning strengths satisfying condition (4).

For ER networks, pinning density *n*_*D*_ increases with network size *N* (Fig. 2a), which can be related to the fact that the number of links in such networks increases with *N* as *N*^2^ for a constant wiring probability *p*. Given that the number of driver nodes in our control strategy depends on the number of links, we expect *N*_*D*_/*N*^2^ = *n*_*D*_/*N* vs. *N* to be constant (see the lower inset of Fig. 2a). We also find that *n*_*D*_ saturates at about 30% for large wiring probabilities *p* (see the upper inset of Fig. 2a). Note that other final-state-oriented control strategies, for instance the feedback vertex set (FVS) control scheme^24^, require a two- to three-fold higher pinning density (from which one finds 75% for *p* = 0.03, 89% for *p* = 0.175, and 93% for *p* = 0.3).

For SF networks, pinning density *n*_*D*_ is independent of the network size *N* and takes on values between 3% – 6% (Fig. 2b). For such networks, the FVS control scheme requires 48% of nodes to be controlled, while it amounts to 37% with structural controllability (in Supplementary Information, we report also our findings obtained with the MSF formalism). The probability density of pinning strength *β* depends on the properties of the coupling topology (Fig. 2c). For SF networks, we find *β* to be only marginally influenced by the number of nodes *N*, and control of these networks requires comparably low strengths ($\beta \simeq 2$). For ER networks with for instance *N* = 120, we find an only marginal influence of the wiring probability (here *p* = 0.03, *p* = 0.3, and *p* = 0.8), but control of these networks requires higher pinning strengths $\beta \simeq 20$) in comparison with SF networks.

### Characteristics of driver nodes

**Figure 3:**
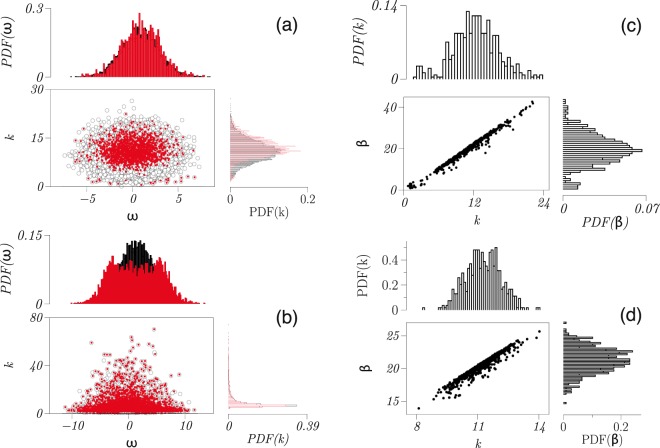
Characteristics of driver nodes in networks of second-order Kuramoto oscillators. Scatter plot of degree *k* and natural frequencies *ω* (deviation from a nominal frequency) of driver nodes (red filled circles) and of all nodes (black circles) in (**a**) ER and (**b**) SF oscillator networks. The results are collected for ER networks (with wiring probability *p* = 0.03) and for SF networks both with *N*∈{50, …, 290}. Scatter plot of degrees *k* and pinning strength *β* of driver nodes in (**c**) ER and (**d**) SF oscillator networks. Here we used our proposed control strategy. The results are also collected for ER networks with wiring probability *p* = 0.03 and for SF networks, but with *N* ∈ {50, …, 150}.

Having characterized important properties of our control approach (pinning density and pinning strength), we next investigate whether driver nodes distinguish themselves by specific characteristics. Our findings obtained from extensive numerical simulations and analyses of oscillatory dynamics (second-order Kuramoto oscillators) on about 1000 ER and SF networks are compiled into Fig. 3. Mean natural frequency of oscillators associated with driver nodes in ER networks equals the one associated with non-driver nodes (with $\langle \omega \rangle \simeq 0$) although with a reduced spread (~2.2). For SF networks, however, natural frequencies of driver node oscillators have a localized distribution around ~4 and ~−4, but the mean degrees of the driver nodes (〈*k*_*D*_〉≃1.5) are smaller in comparison with the mean degree of all nodes ($\langle k\rangle \simeq 4$). These findings demonstrate that properties of driver nodes – namely their natural frequency and their mean degree – are related to the underlying coupling topology.

We also find that the pinning strength *β* is highly correlated to the degree *k* of driver nodes for both ER (with *p* = 0.03) and SF networks of second-order Kuramoto oscillators. The averaged values of degree *k* of driver nodes and pinning strength *β* for both networks are 〈$\langle k\rangle \simeq 11$ and $\langle \beta \rangle \simeq 20$.

### Controlling second-order kuramoto oscillators on a power grid

**Figure 4:**
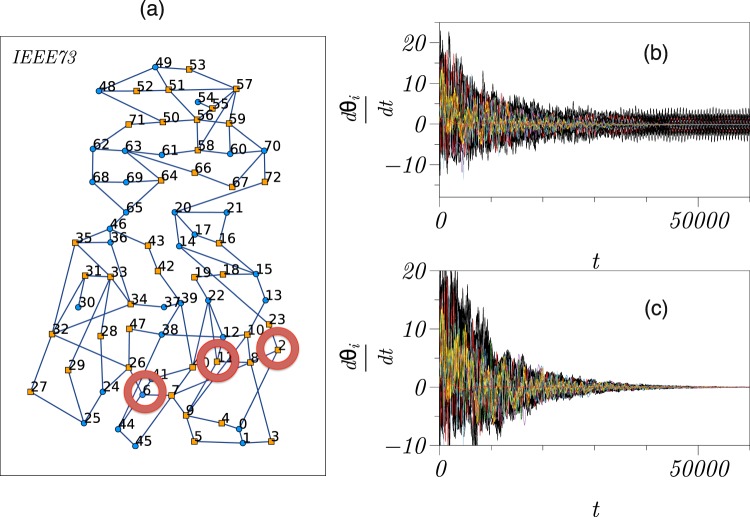
Control of second-order Kuramoto oscillators coupled onto a power grid. (**a**) Power-grid of the IEEE Reliability Test System (IEEE73) consisting of 33 generators (squares) and 40 consumers (circles). Red circles mark driver nodes identified with our scheme (nodes 2, 6, and 11). (**b**) Frequency dynamics of second-order Kuramoto oscillators coupled onto the power grid with lowered damping constant (from *α* = 2 to *α* = 0.8) and starting from some random initial condition from which it does not achieve the fully synchronised state. (**c**) Frequency dynamics of the controlled power grid with *N*_*D*_ = 3 driver nodes starting from the same initial condition. Pinning strengths of driver nodes amounted to *β*_*i*_∈{51, 47, 101}, *i* ∈ {2, 6, 11}. The power grid reaches the synchronised state after about 60000 integration time steps (4th-order Runge-Kutta method with step size *dt* = 10^−3^).

We now demonstrate the efficiency of our approach to control oscillators networks with realistic coupling topologies. To this end, we place second-order Kuramoto oscillators onto the relatively simple, but representative network of the IEEE Reliability Test System^42,43^ (Fig. 4a, Supplementary Information), which is often used to investigate stability of real-world power grids, and contrast our findings to those obtained with the FVS control scheme.

The second-order Kuramoto oscillators on this power grid will synchronise, if we choose a sufficiently high damping constant *α* (Eq. 1; Table S1 in Supplementary Information). Upon reducing the damping constant (from *α* = 2 to *α* = 0.8) and keeping other network properties unchanged, the grid will not synchronise (Fig. 4b). Using our driver node identification scheme, we find that only *N*_*D*_ = 3 out of 73 nodes (4%) are required to control the grid, and when adding the control function with the form $-{\beta }_{i}({\tilde{\nu }}_{i}-{\tilde{\nu }}_{i}^{\ast })$ to the dynamics of these driver nodes, the grid converges to the fully synchronised state (Fig. 4c).

The FVS control scheme requires 53% of the grid’s nodes to be controlled. In general, sets of driver nodes identified with either scheme do not fully overlap, and for the example presented above, only two driver nodes identified with our scheme are also driver nodes identified with the FVS scheme.

We note that the pinning strength *β* is related to parameters and characteristics of the power grid (Table S1, Supplementary Information) and for a given damping constant (dissipation coefficient) can be adjusted by modifying either the adjacency matrix or the reactance of connections linked to a driver node.

With an eye on applications to real power grids, we now aim at controlling the IEEE Reliability Test System under the impact of noise, an essential ingredient for power grids in view of increasing renewable energy sources^44–52^. Here, we consider the dynamics of the rotors (Supplementary Information, Eq. S.16) and add a noise term only to the generators (i.e., rotors with positive natural frequency) as follows

5 $${\ddot{\theta }}_{i}(t)={\omega }_{i}+{\eta }_{i}(t)-\alpha {\dot{\theta }}_{i}(t)+\mathop{\sum }\limits_{k=1}^{N}\,{\lambda }_{ik}\,\sin \,({\theta }_{i}(t)-{\theta }_{k}(t)),$$

where *η*_*i*_(*t*) is an uncorrelated white noise chosen randomly between *ω*_*i*_(1 − *γ*/2) and *ω*_*i*_(1 + *γ*/2) with uniform distribution (Supplementary Information). Here, *γ*∈{0, 2} determines the standard deviation of the noise, which is temporally and spatially uncorrelated (w.r.t. the network).

The added noise *η*_*i*_(*t*) does not depend directly on the state variables of the system, and accordingly, does not appear in the Jacobian matrix, similar to the natural frequency *ω*_*i*_. Therefore, it can be considered as the momentary natural frequency *ω*_*i*_ of generator *i* when added to the constant power input *ω*_*i*_. However, changing *ω*_*i*_ at every time step changes the fixed point of the system as well as its corresponding Jacobian matrix. Since our driver-node-identification scheme is based on the eigenvalues of the Jacobian matrix at the given fixed point, one should run the proposed scheme at every time point. In practice, however, one can find the sets of driver nodes for a given number of noise realisations where at each realisation, the natural frequency of each node is drawn from the aforementioned distribution. Pinning the union of these sets of the driver nodes would be sufficient for stabilising the system when the pinning strength of every node is equal to the maximum of the pinning strengths for that node among all realisations. It should be noted that the noise in the natural frequency is directly related to the noise in the power input of the generators (see Supplementary Information for more details).

**Figure 5:**
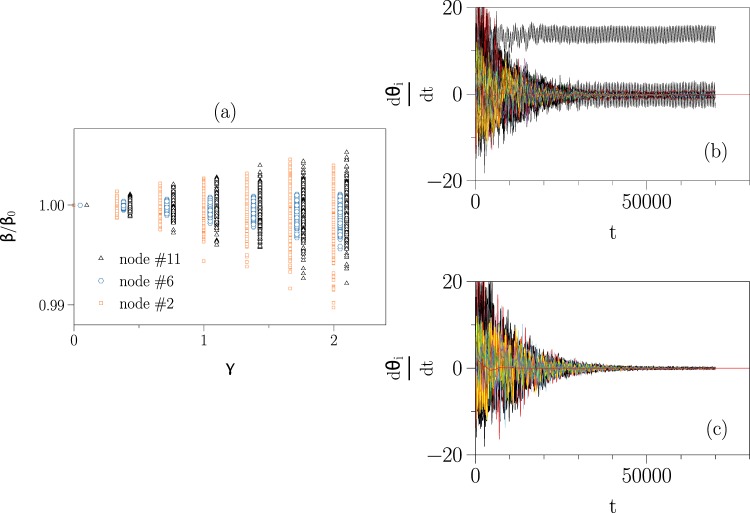
Control of noisy second-order Kuramoto oscillators coupled onto a power grid. (**a**) Normalised pinning strengths *β* for the three identified driver nodes of the IEEE73 test system (see Fig. 4) under the impact of uncorrelated uniformly distributed white noise with different standard deviation as determined by *γ* (*β*_0_ denotes the pinning strength of the noise-free system). Data for nodes 6 and 11 are slightly shifted to the right for the sake of visibility. (**b**) Noisy frequency dynamics (*γ* = 0.1) of second-order Kuramoto oscillators coupled onto the IEEE73 test system starting from some random initial condition from which it does not achieve the fully synchronised state. (**c**) Frequency dynamics of the controlled noisy power grid with *N*_*D*_ = 3 driver nodes starting from the same initial condition. Pinning strengths of driver nodes amounted to *β*_*i*_∈{53, 49, 103}, *i*∈{2, 6, 11}. A convergence of the power grid frequency is attained after about 50000 integration time steps (2nd-order stochastic Runge-Kutta method with step size *dt* = 10^−3^).

Here, we identify driver nodes for 500 realisations of the noise. For each realisation, the identified driver nodes coincide with the three nodes identified for the noise-free system. Nevertheless, the required pinning strengths (normalized to the respective values from the noise-free system) attain a wider distribution when the standard deviation of the noise is increased (Fig. 5), as expected. If we pin the union of sets of driver nodes for each realisation with the maximum pinning strength obtained from the different realisations, we can control the noisy system as shown in Fig. 5.

### Controlling networks of complex multi-dimensional dynamical systems

Extending our results for relatively simple oscillators, we finally demonstrate the efficiency of our approach to control more complicated dynamical systems. As an example, we consider networks of *N* coupled Rössler oscillators, where oscillator *i* evolves according to

6 $$\frac{d}{dt}{{\bf{x}}}^{i}=F({{\bf{x}}}^{i})-\sigma \mathop{\sum }\limits_{j=1}^{N}\,{g}_{ij}H({{\bf{x}}}^{j})$$

with

$${{\bf{x}}}^{i}=[\begin{array}{c}{x}_{1}^{i}\\ {x}_{2}^{i}\\ {x}_{3}^{i}\end{array}],\,F({{\bf{x}}}^{i})=[\begin{array}{c}-{x}_{2}^{i}-{x}_{3}^{i}\\ {x}_{1}^{i}+a{x}_{2}^{i}\\ b+({x}_{1}^{i}-c){x}_{3}^{i}\end{array}],\,H({{\bf{x}}}^{i})=[\begin{array}{c}{x}_{1}^{i}\\ 0\\ {x}_{3}^{i}\end{array}].$$

**x**^*i*^ denotes the oscillator’s internal state and *σ* is the global coupling strength. We fix the model’s control parameter (*a* = −0.2, *b* = 0.2, *c* = 5.4) so that the system either approaches its fixed point (here fully synchronised state) or diverges depending on initial conditions. Accordingly, the aim of our control scheme will be to steer the system to the fully synchronised state and to prevent divergence (in Supplementary Information, we present an alternative iterative scheme for identifying driver nodes if the desired state is a chaotic attractor).

Oscillators are coupled through their *x*_1_ and *x*_3_ components via the coupling function *H*, and *g*_*ij*_ is the Laplacian matrix associated with the weighted adjacency matrix **A** of the network as ${g}_{ij}={\delta }_{ij}({\sum }_{l\mathrm{=1}}^{N}{a}_{il})-{a}_{ij}$, with *a*_*ij*_ denoting elements of **A**.

To control this network, we derive a term (Supplementary Information) of the form $$-{\beta }_{i}({x}_{3}^{i}-{x}_{3}^{i\ast })+{\beta }_{i}{\alpha }_{i}$$$$({x}_{1}^{i}-{x}_{1}^{i\ast })+{\beta }_{i}{\alpha }_{i}\mathrm{(1}+a)({x}_{2}^{i}-{x}_{2}^{i\ast })$$ that – when added to the equation for $$\frac{d}{dt}{{x}_{3}}^{i}$$ (see Eq. (6)) – enables us to move the eigenvalues of the corresponding Jacobian to the left half-plane of the complex plane. Here $${x}_{j}^{i\ast }$$ (*j*∈{1, 2, 3}) are the stationary solutions of Eq. (6). Also, $${\alpha }_{i}\mathrm{=(2}+a\mathrm{)[2}+\sigma {k}_{i}^{{\rm{in}}}]$$, and $${k}_{i}^{{\rm{in}}}={\sum }_{j}{a}_{ij}$$ is the sum of weights connected to node *i*. The pinning strength *β*_*i*_ of node *i* satisfies the following relation

7 $${\beta }_{i} > {\alpha }_{i}-c+{\tilde{x}}_{1}^{\ast i}-\mathrm{(1}+a){\tilde{x}}_{2}^{\ast i}+{\mathscr{J}},$$

where the term ${\mathscr{J}}$ is related to the Jacobian of the controlled system (Supplementary Information). Note that the suggested control function depends on both, parameters controlling the dynamics (*a* and *σ*) and on properties of the underlying connection topology (${k}_{i}^{{\rm{in}}}$).

**Figure 6:**
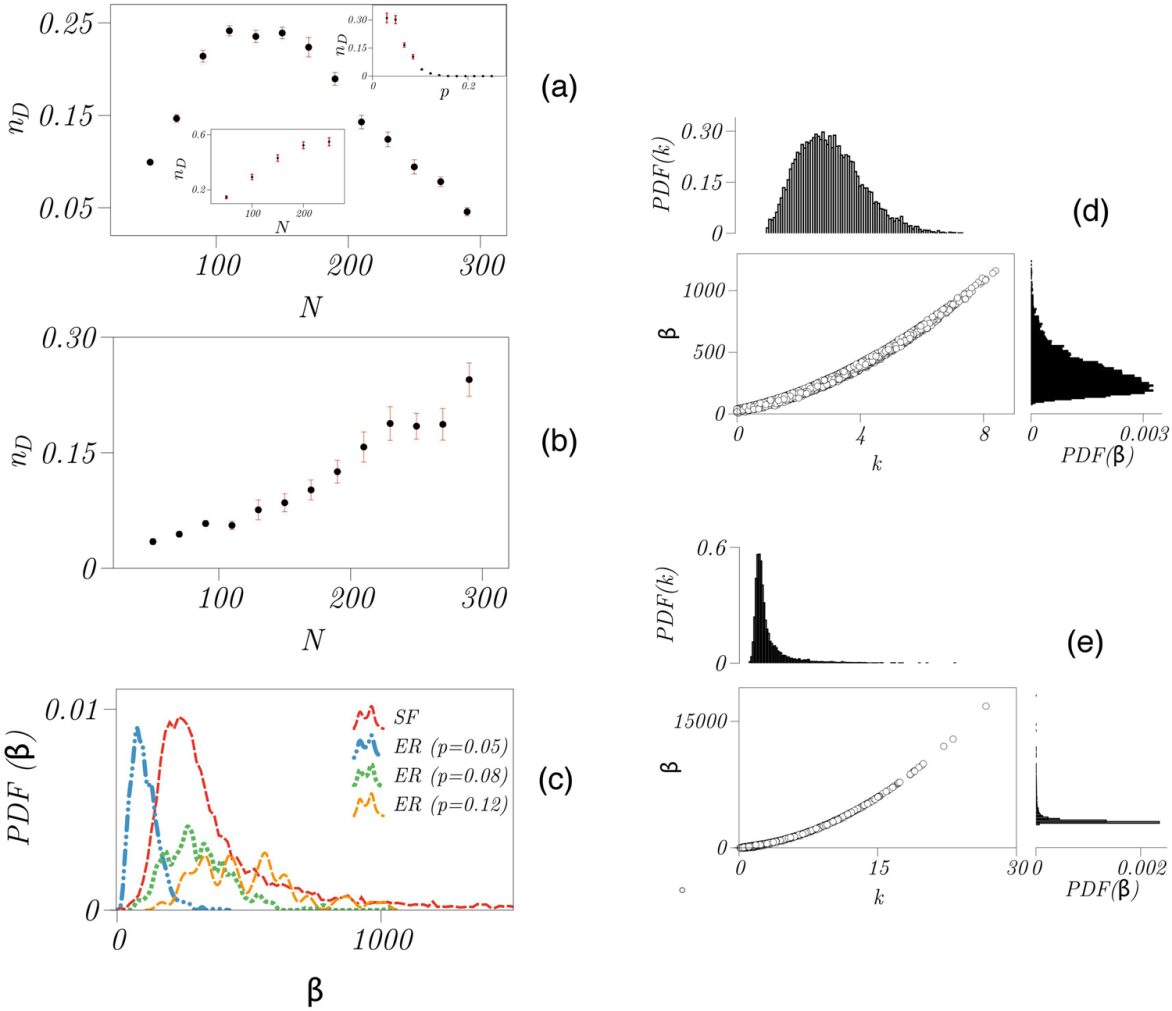
Impact of network characteristics on pinning density and strength for networked Rössler oscillators and characteristics of driver nodes. Dependence of pinning density *n*_*D*_ (fraction of driver nodes) on network size *N* for (**a**) Erdős-Rényi (ER) and (**b**) scale-free (SF) oscillator networks (global coupling strength *σ* = 0.06). For Erdős-Rényi networks, *n*_*D*_ (for *p* = 0.3) decreases slowly for large *N* depending on the wiring probability *p*. For the Watts-Strogatz networks, *n*_*D*_ increases with *N* and also decreases with *p* (see insets in upper panel). Means and error bars from 10 realisations of networks. (**c**) Probability distribution functions (PDF) of pinning strengths *β* required to control (**d**) scale-free (SF) networks and (**e**) Erdős-Rényi (ER) networks with different wiring probabilities *p* and depending on the degree *k* of driver nodes. For each of the 100 realisations of networks each with the number of nodes *N* ∈ {50, …, 290} we chose the minimum pinning strengths satisfying condition (7).

We now proceed as above to identify sets of driver nodes for ER and SF networks with *N* nodes and show in Fig. 6 how pinning density depends on network size *N*. In the case of ER networks, and in contrast to the networks of second-order Kuramoto oscillators, *n*_*D*_ does not increase monotonically with increasing *N*, however, a maximum can be observed at *N* ≈ 130. Moreover, *n*_*D*_ decreases monotonically with increasing the wiring probability *p*, which again contrasts our findings for the networks of second-order Kuramoto oscillators.

The existence of the maximum in the dependence of *n*_*D*_ on *N* is related to the fact that the pinning density is affected by two variables – the mean degree and the network size. One can speculate that for small values of *N*, the impact of the network size dominates and leads to an increase of *n*_*D*_. Likewise, for larger values of *N*, the impact of the mean degree leads to a decrease of *n*_*D*_. To verify this hypothesis, one can study the dependence of *n*_*D*_ vs *N* with a fixed mean degree. The Erdős-Rényi networks could not be used for this purpose since with a fixed wiring probability *p*, the mean degree increases with network size *N*. Accordingly, we use Watts-Strogatz (WS) networks (here with a rewiring probability of 0.9 and with 4 “nearest neighbors”) which are random, sparse networks with a fixed mean degree. For such networks, *n*_*D*_ indeed increases with *N* (lower inset in Fig. 6a). Therefore, by taking into account the decreasing behavior of *n*_*D*_ with an increasing wiring probability *p* (and therefore increasing mean degree; upper inset in the Fig. 6a), and increasing of *n*_*D*_ with *N*, we can understand the existence of the aforementioned maximum for ER networks. Finally, in the case of SF networks, we observe *n*_*D*_ to increase monotonically with network size, in contrast to the SF networks of second-order Kuramoto oscillators.

Investigating how the probability distribution functions of pinning strengths *β* depend on properties of the network topology (Fig. 6c), we observe in ER networks that both the mean value and the variance of the distribution increase with increasing the wiring probability. In contrast, control of SF networks requires smaller pinning strengths.

As with the networks of second-order Kuramoto oscillators, we observe again that the pinning strength *β* is highly correlated to the node degree *k*, for both SF and ER networks (Fig. 6d,e), however, the correlation points here to a nonlinear relationship. The averaged values of degree *k* of driver nodes for both networks are $\langle k\rangle \simeq 3$, however, the averaged pinning strength *β* are $\langle \beta \rangle \simeq 260$ and $\langle \beta \rangle \simeq 540$ for ER and SF networks, respectively.

## Discussion

Complex systems as diverse as the brain, power grids, food webs, social systems, and even the climate are often modeled as networks of dynamical units, and control of dynamical processes on such networks with complex topologies remains a highly active interdisciplinary field of research^4,53–55^. Control strategies that are solely based on the network’s topological properties^4^ disregard the system’s dynamics, which is usually highly nonlinear. Addressing this issue, we here proposed a straightforward topology-dynamics-based control approach for multi-dimensional complex networked dynamical systems (with and without self-dynamics). Our approach allows to find a suitable control function as well as a finite set of driver nodes (in polynomial time) to steer the system to the desired state. Moreover, the iterative scheme we developed to identify driver nodes can be combined with other control strategies such as the master stability function formalism (MSF)^26^, as discussed in detail in Supplementary Information. In contrast to MSF, our proposed approach can be used to determine the control function and the pinning strength(s) required for controlling complex systems that consist of non-identical components. This makes our approach different from most of the current control strategies.

By extensive numerical simulations and analyses of different multi-dimensional dynamics (second-order Kuramoto and Rössler oscillators) on different networks, we observed properties of driver nodes, such as their degree *k* and the pinning strength *β* to be related to both the underlying coupling topology and dynamics. For instance, our results indicate that in case of second-order Kuramoto dynamics the pinning density *n*_*D*_ mainly depends on the network’s mean degree while in the case of Rössler oscillators, it depends on both the network’s mean degree and size.

Our approach relies on the existence of a fixed point of the system as a desired state, along with full observability and accessibility of the system. We have shown that one can employ our control approach also in cases where the desired state is a nominal trajectory by combining it with the master stability function formalism. It would be interesting to re-express our driver-node-identification scheme using the induced (matrix) norm^10,11,56^.

So far we have demonstrated our control approach using exemplary multi-dimensional dynamical systems, whose equations of motion are known, and using some prototypical network topologies. In a next step, our approach needs to be supplemented with a suitable minimisation of the control energy^55,57^ derived from the corresponding dynamics. Once this has been achieved, we envision data-driven approaches to construct both dynamics and relevant aspects of the coupling^49,58–60^. For these approaches, however, one should take into account that a fully observable system might have poor observability properties^61,62^. Nevertheless, such approaches – together with recently proposed data-driven methods to assess resilience^63^ – would enable control of complex natural^64–67^ and man-made systems^68,69^, where both topology and dynamics play essential roles in their collective behavior.

## Methods

### Linear stability, spectrum of eigenvalues, and Gershgorin circle theorem

A system is linearly stable around a fixed point, if all eigenvalues of the corresponding Jacobian matrix have a negative real part. The vanishing of the positive real part can be verified using the Gershgorin circle theorem^39^, which states that all eigenvalues of a given *N* × *N* matrix M lie within the union of closed Gershgorin disks *D*_*i*_ with *i* = 1, …, *N*. The *i*th disk’s center is positioned at *C*_*i*_ = *M*_*i*,*i*_ and its radius is ${R}_{i}={\sum }_{j\ne i}|{M}_{i,j}|$, where *M*_*i*,*j*_ is an entry of M. Accordingly, to ensure that all eigenvalues lie in the negative half-plane of the complex plane (i.e., all components of dynamical system will approach the fixed point), all Gershgorin disks should be moved to the left half-plane of the complex plane.

### Basic idea to determine the control function

Let us describe a general formalism which can be used to determine a control function for given coupled dynamics. Here, we assume that the system has two-dimensional state variables and that the dynamics is given by

8 $$\dot{x}=f(x,y)\,{\rm{and}}\,\dot{y}=g(x,y),$$

with *f* and *g* being arbitrary and non-linear functions. We assume that the coupled dynamics has a fixed point at (x^*^, y^*^) (by equating *f*(*x*, *y*) = *g*(*x*, *y*) = 0). The Jacobian matrix evaluated at the fixed point reads

9 $${\bf{J}}({x}^{\ast },{y}^{\ast })=[\begin{array}{ll}{\frac{\partial f}{\partial x}|}_{\begin{array}{c}x={x}^{\ast }\\ y={y}^{\ast }\end{array}} & {\frac{\partial f}{\partial y}|}_{\begin{array}{c}x={x}^{\ast }\\ y={y}^{\ast }\end{array}}\\ {\frac{\partial g}{\partial x}|}_{\begin{array}{c}x={x}^{\ast }\\ y={y}^{\ast }\end{array}} & {\frac{\partial g}{\partial y}|}_{\begin{array}{c}x={x}^{\ast }\\ y={y}^{\ast }\end{array}}\end{array}].$$

The center *c*_1_ and radius *r*_1_ of first the Gershgorin disk of this matrix read

10 $${c}_{1}={\frac{\partial f}{\partial x}|}_{\begin{array}{c}x={x}^{\ast }\\ y={y}^{\ast }\end{array}}$$

11 $${r}_{1}=|{\frac{\partial g}{\partial y}|}_{\begin{array}{c}x={x}^{\ast }\\ y={y}^{\ast }\end{array}}|.$$

If *r*_1_ + *c*_1_ ≥ 0, this disk will have some part in the positive half plane of complex plane and consequently, it might cause a positive real part in eigenvalues. Here, we assume that this situation happens, and we determine a control function that allows stabilising the system’s dynamics around its fixed point. We start with a transformation that enables steering the first Gershgorin disk to the left half plane, and the only disk which remains to be controlled will be the second one. We use

12 $$\tilde{x}=x$$

13 $$\tilde{y}=y+h\tilde{x},$$

where $h={\rm{sgn}}(\frac{\partial f}{\partial y}{|}_{\mathop{y={y}^{\ast }}\limits^{x={x}^{\ast }}})$. Accordingly, the dynamics of transformed variables read

14 $$\dot{\tilde{x}}=\frac{d\tilde{x}}{dt}=\frac{\partial \tilde{x}}{\partial x}\frac{dx}{dt}+\frac{\partial \tilde{x}}{\partial y}\frac{dy}{dt}=\dot{x}+0=f(x,y)=f(\tilde{x},\tilde{y}-h\tilde{x})$$

15 $$\dot{\tilde{y}}=\frac{d\tilde{y}}{dt}=\frac{\partial \tilde{y}}{\partial x}\frac{dx}{dt}+\frac{\partial \tilde{y}}{\partial y}\frac{dy}{dt}=h\dot{x}+\dot{y}=g(\tilde{x},\tilde{y}-h\tilde{x}),$$

with the new Jacobian matrix evaluated at the fixed point,

16 $${\bf{J}}({\tilde{x}}^{\ast },{\tilde{y}}^{\ast })=[\begin{array}{ll}{\frac{\partial f}{\partial \tilde{x}}|}_{\begin{array}{c}\tilde{x}={\tilde{x}}^{\ast }\\ \tilde{y}={\tilde{y}}^{\ast }\end{array}} & {\frac{\partial f}{\partial \tilde{y}}|}_{\begin{array}{c}\tilde{x}={\tilde{x}}^{\ast }\\ \tilde{y}={\tilde{y}}^{\ast }\end{array}}\\ {\frac{\partial g}{\partial \tilde{x}}|}_{\begin{array}{c}\tilde{x}={\tilde{x}}^{\ast }\\ \tilde{y}={\tilde{y}}^{\ast }\end{array}} & {\frac{\partial g}{\partial \tilde{y}}|}_{\begin{array}{c}\tilde{x}={\tilde{x}}^{\ast }\\ \tilde{y}={\tilde{y}}^{\ast }\end{array}}\end{array}].$$

One can easily find elements of this matrix to check whether the first Gershgorin disk is placed in the left half plane or not. One finds

17 $$\frac{\partial f}{\partial \tilde{x}}=\frac{\partial f}{\partial x}\frac{\partial x}{\partial \tilde{x}}+\frac{\partial f}{\partial u}\frac{\partial u}{\partial \tilde{x}}=\frac{\partial f}{\partial x}-\frac{\partial f}{\partial y}h$$

18 $$\frac{\partial f}{\partial \tilde{y}}=\frac{\partial f}{\partial x}\frac{\partial x}{\partial \tilde{y}}+\frac{\partial f}{\partial u}\frac{\partial u}{\partial \tilde{y}}=0+\frac{\partial f}{\partial y}$$

where *u* is the second argument of *f* and *g*, which is in fact the variable *y*. Now, the new first Gershgorin disk is centered at ${\tilde{c}}_{1}$ and has radius ${\tilde{r}}_{1}$

19 $${\tilde{c}}_{1}={\frac{\partial f}{\partial x}|}_{\begin{array}{c}\tilde{x}={\tilde{x}}^{\ast }\\ \tilde{y}={\tilde{y}}^{\ast }\end{array}}-{\frac{\partial f}{\partial y}|}_{\begin{array}{c}\tilde{x}={\tilde{x}}^{\ast }\\ \tilde{y}={\tilde{y}}^{\ast }\end{array}}h$$

20 $${\tilde{r}}_{1}=|{\frac{\partial f}{\partial y}|}_{\begin{array}{c}\tilde{x}={\tilde{x}}^{\ast }\\ \tilde{y}={\tilde{y}}^{\ast }\end{array}}|.$$

It is obvious that ${\tilde{c}}_{1}+{\tilde{r}}_{1}=0$ holds if ${\tilde{c}}_{1}$ is negative which is true in practical cases.

Next, we should make sure that the second Gershgorin disk has no positive part. To do so, we first calculate its center ${\tilde{c}}_{2}$ and radius ${\tilde{r}}_{2}$

21 $${\tilde{c}}_{2}={\frac{\partial g}{\partial \tilde{y}}|}_{\begin{array}{c}\tilde{x}={\tilde{x}}^{\ast }\\ \tilde{y}={\tilde{y}}^{\ast }\end{array}}={\frac{\partial g}{\partial y}|}_{\begin{array}{c}x={x}^{\ast }\\ y={y}^{\ast }\end{array}}$$

22 $${\tilde{r}}_{2}=|{\frac{\partial g}{\partial \tilde{x}}|}_{\begin{array}{c}\tilde{x}={\tilde{x}}^{\ast }\\ \tilde{y}={\tilde{y}}^{\ast }\end{array}}|={\frac{\partial g}{\partial x}|}_{\begin{array}{c}\tilde{x}={\tilde{x}}^{\ast }\\ \tilde{y}={\tilde{y}}^{\ast }\end{array}}+{\frac{\partial g}{\partial y}|}_{\begin{array}{c}\tilde{x}={\tilde{x}}^{\ast }\\ \tilde{y}={\tilde{y}}^{\ast }\end{array}}h.$$

Now, if  ${\tilde{c}}_{2}+{\tilde{r}}_{2}\ge 0$, one can add a pinning term of the form of $-\beta (\tilde{y}-{\tilde{y}}^{\ast })$ to the r.h.s. of Eq. 15. The pinning term adds a (−*β*) to the r.h.s. of Eq. 21 and moves the center to the left. To make sure that the Gershgorin disk has no positive real part, *β* should satisfy the f ollowing condition

23 $$\beta \ge {\tilde{c}}_{2}+{\tilde{r}}_{2}.$$

It s hould be noted that adding the term $-\beta (\tilde{y}-{\tilde{y}}^{\ast })$ to Eq. 15 is equivalent to adding −*β*(*y* − y^*^) − *hβ*(*x* − x^*^) to Eq. 8.

### Control function for networks of second-order kuramoto oscillators

The equation of motion of the network of second-order Kuramoto oscillators (Eq. (1)) can be rewritten as

24 $$\begin{array}{rcl}{\dot{\theta }}_{i} & = & {\nu }_{i}\\ {\dot{\nu }}_{i} & = & {\omega }_{i}-\alpha {\nu }_{i}+\mathop{\sum }\limits_{j=1}^{N}\,{\lambda }_{ij}\,\sin \,({\theta }_{j}-{\theta }_{i}),\end{array}$$

for which the Jacobian matrix evaluated at the fixed point reads25*I* is the *N* × *N* unit matrix and entries of matrix M are

26 $${M}_{ij}=(\begin{array}{cc}-\sum _{k}\,{\lambda }_{ik}\,\cos \,({\theta }_{k}^{\ast }-{\theta }_{i}^{\ast }) & i=j\\ {\lambda }_{ij}\,\cos \,({\theta }_{j}^{\ast }-{\theta }_{i}^{\ast }) & {\rm{otherwise}}\mathrm{}.\end{array}$$

It is evident that the first *N* (out of 2*N*) Gershgorin dis ks for this Jacobian are centered at the origin and ha ve unit radii. The corresponding eigenvalues thus may possess a positive real part. To avoid this, we define tw o new variables, ${\tilde{\theta }}_{i}={\theta }_{i}$ and ${\tilde{\nu }}_{i}={\nu }_{i}+{\theta }_{i}$. This change of variables changes the g overn ing equations to (see Eq. (2)):

$$\begin{array}{rcl}{\dot{\tilde{\theta }}}_{i} & = & {\tilde{\nu }}_{i}-{\tilde{\theta }}_{i}\\ {\dot{\tilde{\nu }}}_{i} & = & {\omega }_{i}+(1-\alpha ){\tilde{\nu }}_{i}-(1-\alpha ){\tilde{\theta }}_{i}+\mathop{\sum }\limits_{j=1}^{N}\,{\lambda }_{ij}\,\sin \,({\tilde{\theta }}_{j}-{\tilde{\theta }}_{i}),\end{array}$$

for which the Jacobian matrix evaluated at the fixed point has the following form27

The entries of matrix M are

28 $${M}_{ij}=\{\begin{array}{ll}\alpha -1-\sum _{k}\,{\lambda }_{ik}\,\cos \,({\theta }_{k}^{\ast }-{\theta }_{i}^{\ast }) & j=i-N\\ 1-\alpha  & j=i\\ {\lambda }_{ij}\,\cos \,({\theta }_{j}^{\ast }-{\theta }_{i}^{\ast }) & j < N\,\& \,j\ne i-N\\ 0 & j > N\,\& \,j\ne i.\end{array}$$

Here, ${\theta }_{i}^{\ast }$ denotes the equilibrium (or resting) point of node *i*, i.e., the value of the state variable for which ${\dot{\tilde{\theta }}}_{i}={\dot{\tilde{\nu }}}_{i}=0$, and it may be a stable or an unstable fixed point. It is easy to see that adding a term of the form $-{\beta }_{i}({\tilde{\nu }}_{i}-{\tilde{\nu }}_{i}^{\ast })$ to Eq. (2) enables us to move the eigenvalues of the Jacobian to t he left half-plane of the complex plane, if the pinning strength *β*_*i*_ satisfies condition (4).

## Supplementary information

Supplementary Information

## Data Availability

The data and code utilized in this study are available from the corresponding author upon reasonable request.
