# Supplementary material for: A topology-dynamics-based control strategy for multi-dimensional complex networked dynamical systems
Source: Sci Rep. 2019 Dec 27;9:19831. doi: 10.1038/s41598-019-56259-4 (PMC6934796; doi:10.1038/s41598-019-56259-4)
Supplement: Supplementary file 1 — Supplementary Information [file 41598_2019_56259_MOESM1_ESM.pdf]

Supplementary Information for "A topology-dynamics-based control strategy for multi-dimensional complex networked dynamical systems"

Mohammadreza Bahadorian[1], Hamidreza Alimohammadi[1], Tahereh Mozaffari[1], Mohammad Reza Rahimi Tabar[1], Joachim Peinke[2], Klaus Lehnertz[3,4,5,*]

[1]Department of Physics, Sharif University of Technology, Tehran 11155-9161, Iran

[2]Institute of Physics and ForWind, Carl von Ossietzky University of Oldenburg, Carl-von-Ossietzky-Straße 9–11, 26111 Oldenburg, Germany

[3]Department of Epileptology, University of Bonn, Venusberg Campus 1, 53127 Bonn, Germany

[4]Helmholtz-Institute for Radiation and Nuclear Physics, University of Bonn, Nussallee 14–16, 53115 Bonn, Germany

[5]Interdisciplinary Center for Complex Systems, University of Bonn, Brühler Straße 7, 53175 Bonn, Germany

[*]To whom correspondence should be addressed; E-mail: klaus.lehnertz@ukbonn.de

Control function for networks of Rössler oscillators

The equations of motion of the networks of Rössler oscillators read

$$\begin{aligned}
\dot{x}_1^i &= -x_2^i - x_3^i + \sigma \sum_j a_{ij}(x_1^j - x_1^i) \\
\dot{x}_2^i &= x_1^i + a x_2^i \\
\dot{x}_3^i &= b + (x_1^i - c)x_3^i + \sigma \sum_j a_{ij}(x_3^j - x_3^i),
\end{aligned} \qquad \text{(S.1)}$$

and the corresponding Jacobian matrix evaluated at the fixed point is given by

$$\mathbf{J} = \begin{array}{c} \\ \end{array} \begin{array}{ccc} x_1 & x_2 & x_3 \\ \end{array}$$

$$\mathbf{J} = \left[\begin{array}{c|c|c} \mathbf{L} & -\mathbf{I} & \mathbf{I} \\ \hline \mathbf{I} & a\mathbf{I} & 0 \\ \hline \mathrm{diag}(x_3^{*i}) & 0 & \mathbf{M} \end{array}\right] \begin{array}{c} \dot{x}_1 \\ \dot{x}_2 \\ \dot{x}_3 F \end{array}$$

(columns labelled x_1, x_2, x_3)

where $\mathbf{I}$ is the unit matrix, and the matrices $\mathbf{M}$ and $\mathbf{L}$ have entries

$$M_{ij} = \begin{cases} x_1^{*i} - c - \sigma k_i^{\text{in}} & i = j \\ \sigma a_{ij} & i \neq j \end{cases} \quad , \quad L_{ij} = \begin{cases} -\sigma k_i^{\text{in}} & i = j \\ \sigma a_{ij} & i \neq j \end{cases} \; ,$$

and where $k_i^{\text{in}} = \Sigma_j a_{ij}$, and x_j^{*i} ($j \in \{1, 2, 3\}$) are the stationary solutions of equation (S.1).

The part of the Jacobian matrix which corresponds to the $\partial_{1,2,3}\dot{x}_2^i$ has positive diagonal elements and this can cause instability. With the following change of variables

$$\begin{aligned}
\tilde{x}_1^i &= x_1^i + (1+a)x_2^i \\
\tilde{x}_2^i &= x_2^i \\
\tilde{x}_3^i &= x_3^i - \alpha_i \tilde{x}_1^i,
\end{aligned} \qquad \text{(S.2)}$$

where $\alpha_i = 2[1 + (1 + a)(1 + \sigma k_i^{\text{in}})]$, the problematic part of the Jacobian vanishes. With

equations (S.2), we now find

$$
\begin{aligned}
\dot{\tilde{x}}_1^i &= \dot{x}_1^i + (1+a)\dot{x}_2^i = -x_2^i - x_3^i + \sigma \sum_j a_{ij} x_1^j - \sigma k_i^{\text{in}} x_1^i + (1+a)(\tilde{x}_1^i - \tilde{x}_2^i) \\
&= -(2 + a - (1+a)\sigma k_i^{\text{in}})\tilde{x}_2^i + (1 + a - \sigma k_i^{\text{in}})\tilde{x}_1^i + \sigma \sum_j a_{ij}(\tilde{x}_1^j - (1+a)\tilde{x}_2^j) - \tilde{x}_3^i \\
&- 2[1 + (1+a)(1+\sigma k_i^{\text{in}})]\tilde{x}_1^i \\
&= -(2 + a + (1+a)\sigma k_i^{\text{in}})\tilde{x}_2^i - [(3+a) + (3+2a)\sigma k_i^{\text{in}}]\tilde{x}_1^i \\
&+ \sigma \sum_j a_{ij}(\tilde{x}_1^j - (1+a)\tilde{x}_2^j) - \tilde{x}_3^i
\end{aligned} \tag{S.3}
$$

$$
\begin{aligned}
\dot{\tilde{x}}_2^i &= \dot{x}_2^i = x_1^i + a x_2^i = \tilde{x}_1^i - (1+a)\tilde{x}_2^i + a\tilde{x}_2^i \\
&= -\tilde{x}_2^i + \tilde{x}_1^i
\end{aligned} \tag{S.4}
$$

$$
\begin{aligned}
\dot{\tilde{x}}_3^i &= \dot{x}_3^i - \alpha_i \dot{\tilde{x}}_1^i \\
&= b + (x_1^i - c)x_3^i + \sigma \sum_j a_{ij} x_3^j - \sigma k_i^{\text{in}} x_3^i - \alpha_i \dot{\tilde{x}}_1^i \\
&= b + (\tilde{x}_1^i - (1+a)\tilde{x}_2^i - c - \sigma k_i^{\text{in}})[\tilde{x}_3^i + \alpha_i \tilde{x}_1^i] \\
&+ \sigma \sum_j a_{ij}(\tilde{x}_3^j + \alpha_j \tilde{x}_1^j) - \alpha_i(2 + a + (1+a)\sigma k_i^{\text{in}})\tilde{x}_2^i \\
&- \alpha_i[3 + a + (3+2a)\sigma k_i^{\text{in}}]\tilde{x}_1^i - \alpha_i \tilde{x}_3^i + \alpha_i \sigma \sum_j a_{ij}(\tilde{x}_1^j - (1+a)\tilde{x}_2^j).
\end{aligned} \tag{S.5}
$$

Adding the control function $\beta_i(\tilde{x}_3^i - \tilde{x}_3^{i*})$ to the equation (S.5) leads to the Jacobian matrix evaluated at the fixed point

$$
\tilde{\mathbf{J}} = \begin{array}{c} \begin{array}{ccc} \tilde{x}_1 & \tilde{x}_2 & \tilde{x}_3 \end{array} \\ \left[\begin{array}{c|c|c} \boldsymbol{\mathcal{A}} & \boldsymbol{\mathcal{B}} & -\mathbf{I} \\ \hline \mathbf{I} & -\mathbf{I} & 0 \\ \hline \boldsymbol{\mathcal{C}} & \boldsymbol{\mathcal{D}} & \boldsymbol{\mathcal{E}} \end{array}\right] \end{array} \begin{array}{c} \dot{\tilde{x}}_1 \\ \dot{\tilde{x}}_2 \\ \dot{\tilde{x}}_3 \end{array}
$$

where $\mathbf{I}$ is the unit matrix, and the matrices $\boldsymbol{\mathcal{A}}$ - $\boldsymbol{\mathcal{E}}$ have entries

$$\mathcal{A}_{ij} = \begin{cases} -\left[(3+a) + (3+2a)\sigma k_i^{\text{in}}\right] & i = j \\ \sigma a_{ij} & i \neq j, \end{cases}$$

$$\mathcal{B}_{ij} = \begin{cases} -(2+a+(1+a)\sigma k_i^{\text{in}}) & i = j \\ -\sigma a_{ij}(1+a) & i \neq j, \end{cases}$$

$$\mathcal{C}_{ij} = \begin{cases} \tilde{x}_3^{*i} + \alpha_i \tilde{x}_1^{*i} + \alpha_i(\tilde{x}_1^{*i} - (1+a)\tilde{x}_2^{*i} - c) & \\ \quad - \alpha_i\left[a+3+(4+2a)\sigma k_i^{\text{in}}\right] & i = j \\ \sigma a_{ij}(\alpha_i + \alpha_j) & i \neq j, \end{cases}$$

$$\mathcal{D}_{ij} = \begin{cases} -(1+a)\left[\tilde{x}_3^{*i} + \alpha_i \tilde{x}_1^{*i}\right] - \alpha_i\left[2+a+(1+a)\sigma k_i^{\text{in}}\right] & i = j \\ -\alpha_i \sigma (1+a) a_{ij} & i \neq j, \end{cases}$$

and

$$\mathcal{E}_{ij} = \begin{cases} -\alpha_i - c + \tilde{x}_1^{i*} - (1+a)\tilde{x}_2^{i*} - \sigma k_i^{\text{in}} - \beta_i & i = j \\ \sigma a_{ij} & i \neq j. \end{cases}$$

Centers (C_k) and radii (R_k) of the Gershgorin disks of the related Jacobian $\tilde{\mathbf{J}}$ are

$$\begin{aligned} C_1 &= -(3+a) - (3+2a)\sigma k_i^{\text{in}}, \\ R_1 &= +(3+a) + (3+2a)\sigma k_i^{\text{in}}, \\ C_2 &= -1, \\ R_2 &= 1, \\ C_3 &= \mathcal{E}_{ii}, \\ R_3 &= \sum_j (|\mathcal{C}_{ij}| + |\mathcal{D}_{ij}| + |\mathcal{E}_{ij}|) \end{aligned}$$

where $C_1 + R_1 = 0$, $C_2 + R_2 = 0$ and the pinning strength β_i of node i satisfies the following relation

$$\beta_i > \alpha_i - c + \tilde{x}_1^{*i} - (1+a)\tilde{x}_2^{*i} + \sum_j (|\mathcal{E}_{ij}| + |\mathcal{D}_{ij}| + |\mathcal{C}_{ij}|) + \epsilon. \tag{S.6}$$

In our numerical simulations, we set $\epsilon = 0.2$. We note that adding the control function $\beta_i(\tilde{x}_3^i - \tilde{x}_3^{i*})$ to equation (S.5) is equivalent to adding in equation (S.1) the following control function to

$\dot{x}_3^i$

$$-\beta_i(x_3^i - x_3^{i*}) + \beta_i\alpha_i(x_1^i - x_1^{i*}) + \beta_i\alpha_i(1+a)(x_2^i - x_2^{i*}),$$

with $\alpha_i = 2[1 + (1+a)(1+\sigma k_i^{\text{in}})]$, and β_i satisfies relation (S.6).

Identifying driver nodes for networks of second-order Kuramoto oscillators with the master stability function formalism

We consider a network of N coupled oscillators, for which the dynamics is given by

$$\frac{d}{dt}\mathbf{x}^i(t) = F(\mathbf{x}^i(t)) - \sigma\sum_{j=1}^{N} g_{ij}H(\mathbf{x}^j(t)). \tag{S.7}$$

Here, the m-dimensional vector $\mathbf{x}^i(t)$ determines the internal state of the node i at time t, σ is the global coupling strength for the network, $F(\mathbf{x}^i)(F : \mathbb{R}^m \to \mathbb{R}^m)$ is the self-action of the dynamics and function $H : \mathbb{R}^m \to \mathbb{R}^m$ couples the oscillators dynamically. The matrix $\mathbf{G}$ is the Laplacian matrix associated with the adjacency matrix $\mathbf{A}$ of the network as $g_{ij} = \delta_{ij}\left(\sum_{k=1}^{N} a_{ik}\right) - a_{ij}$.

In order to control such a network, we shall consider a pinning control strategy. We consider feedback controllers of the form $u_i(t) = \sigma[p_i(\mathbf{s}(t)) - p_i(\mathbf{x}^i(t))]$ with $p_i(\mathbf{x}^i(t)) = \beta_i H(\mathbf{x}^i(t))$ being the pinning function that determines the control input of node i with pinning strength β_i and add the controlling term $u_i(t)$ to the dynamics (equation (S.7)) (*1*). Minor rearrangement of equation (S.7) will lead to the new dynamics of a homogeneous form like in equation (S.7)

$$\frac{d}{dt}\mathbf{x}^i(t) = F(\mathbf{x}^i(t)) - \sigma\sum_{j=1}^{N+1} m_{ij}H(\mathbf{x}^j(t)) \tag{S.8}$$

where $m_{ij} = g_{ij} + \beta_i\,\delta_i\,(\delta_{i,j} - \delta_{N+1,j})$ with $\delta_i = 1$ if the node i is pinned and $\delta_i = 0$ otherwise, is an entry of the effective coupling matrix $\mathbf{M}$ having the same characteristics as the zero-sum

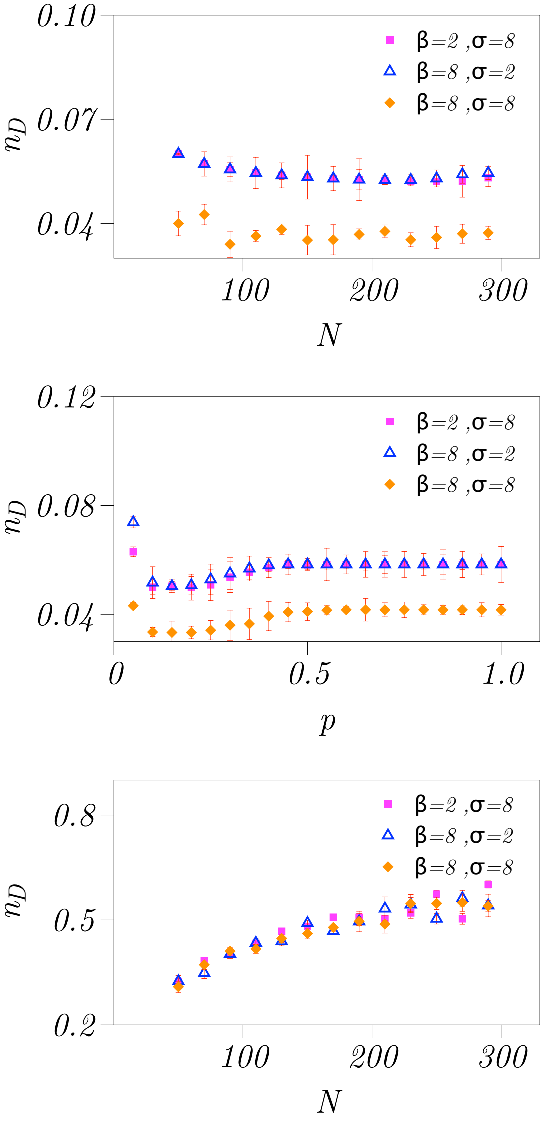

Figure S1: Pinning density n_D (fraction of driver nodes) derived with the MSF-based control scheme versus N and p for all combinations of $\{\sigma, \beta\} = \{2, 8\}$. Top: Erdős-Rényi networks (wiring probability $p = 0.3$), middle: Erdős-Rényi networks with different wiring probabilities for $N = 120$, bottom: scale-free networks of second-order Kuramoto oscillators. Results are averaged over 100 realizations at each point. See Figure 2 in main text.

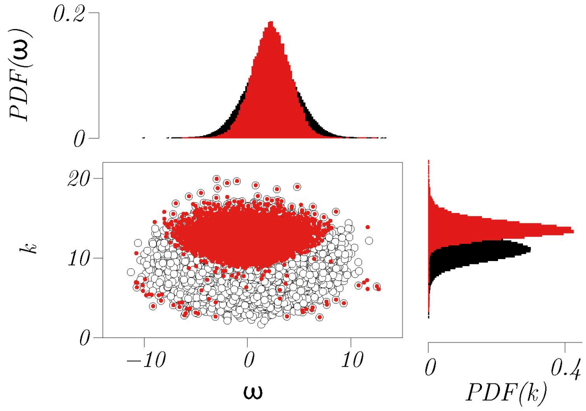

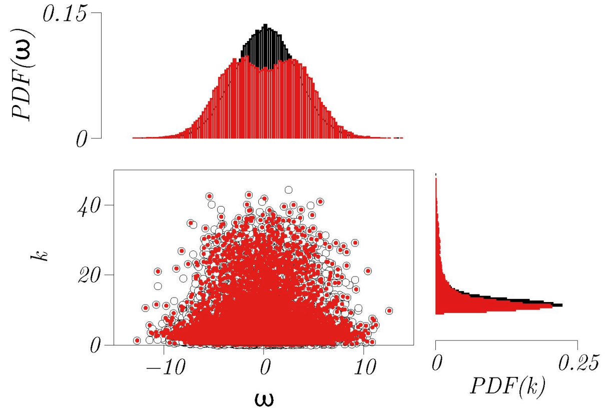

Figure S2: Scatter plot of degrees k and natural frequencies ω of driver nodes (red filled circles) derived with the MSF-based control scheme and of all other nodes (black circles) in ER (p=0.3) (top) and SF networks (bottom) of second-order Kuramoto oscillators. See Figure 3 (parts a and b) in main text.

matrix $\mathbf{G}$. The additional $N+1$-th node is the leader node with the dynamics

$$\frac{d}{dt}\mathbf{s}(t) = F(\mathbf{s}(t)) \tag{S.9}$$

determining the final desired state towards which the whole system is controlled and it is directly connected to the pinned sites of the network, structurally.

By linearizing equation (S.8) around the desired state $\mathbf{s}(t)$ and applying the master stability function (MSF) formalism (*1, 2*) we find

$$\frac{d}{dt}\xi_i(t) = \left[\boldsymbol{\mathcal{F}}(\mathbf{s}(t)) - \sigma\lambda_i\boldsymbol{\mathcal{H}}(\mathbf{s}(t))\right]\xi_i(t) \tag{S.10}$$

where ξ_i is the eigenmode associated with the eigenvalue λ_i of the extended control matrix $\mathbf{M}$, the stability of which for all eigenvalues will guarantee the stability of the solution $\mathbf{s}(t)$ for the controlled network. $\boldsymbol{\mathcal{F}}(\mathbf{s}(t))$ and $\boldsymbol{\mathcal{H}}(\mathbf{s}(t))$ are the Jacobian matrices (evaluated at the fixed point) of $F(\mathbf{x}(t))$ and $H(\mathbf{x}(t))$ for $\mathbf{x}(t) = \mathbf{s}(t)$.

In order to identify a control scheme for networks of second-order Kuramoto oscillators, we linearize equation (1) (main text) in the neighborhood of the desired state $(\boldsymbol{\theta}^*, \boldsymbol{\nu}^*)$, and obtain

$$\boldsymbol{\theta}^* = \mathbf{G}^{\dagger}\boldsymbol{\omega},\ \boldsymbol{\nu}^* = 0 \tag{S.11}$$

with $\mathbf{G}^{\dagger}$ being the pseudo-inverse of the Laplacian of the main network and the vector $\boldsymbol{\omega}$ containing the natural frequencies of all subsystems. This linearization leads to

$$\begin{aligned}
\dot{\delta\theta_i} &= \delta\nu_i \\
\dot{\delta\nu_i} &= -\alpha\delta\nu_i + \sum_{j\neq i}^{N}\lambda_{ij}\cos(\theta_j^* - \theta_i^*)\delta\theta_j - \delta\theta_i\sum_{j\neq i}^{N}\lambda_{ij}\cos(\theta_j^* - \theta_i^*),
\end{aligned}$$

which can be written as

$$\begin{pmatrix}\dot{\delta\theta_i}\\ \dot{\delta\nu_i}\end{pmatrix} = \begin{pmatrix}0 & 1\\ 0 & -\alpha\end{pmatrix}\begin{pmatrix}\delta\theta_i\\ \delta\nu_i\end{pmatrix} - \sigma\sum_{j=1}^{N}\tilde{g}_{ij}\begin{pmatrix}0 & 0\\ 1 & 0\end{pmatrix}\begin{pmatrix}\delta\theta_j\\ \delta\nu_j\end{pmatrix},$$

in which the entries of the zero-sum matrix $\tilde{\mathbf{G}}$ read

$$\tilde{g}_{ij} = \begin{cases} \sum_{j\neq i}^{N} \frac{1}{\sigma}\lambda_{ij}\cos(\theta_j^* - \theta_i^*) & i = j, \\ -\frac{1}{\sigma}\lambda_{ij}\cos(\theta_j^* - \theta_i^*) & i \neq j. \end{cases} \tag{S.12}$$

Note that we have taken out the parameter σ to study the effect of the coupling strength alongside with the pinning strength β while leaving the topological characteristics unchanged. Also α is the damping constant – here taken to be $\alpha = 2$ – and λ_{ij} are the coupling strengths. We can see that how self-action and coupling functions reveal themselves as constant matrices, i.e.,

$$F = \begin{pmatrix} 0 & 1 \\ 0 & -\alpha \end{pmatrix},\ H = \begin{pmatrix} 0 & 0 \\ 1 & 0 \end{pmatrix}.$$

We also note that despite the elimination of natural frequencies ω_i in the linearized dynamics, ω_i will still play a role implicitly in the coordinates of the desired state (θ^*, ν^*), as mentioned before.

We apply the MSF-based control scheme, taking the pinning function in the dynamics of the i-th node to be just like what was stated earlier as $u_i(t) = \sigma[p_i(\mathbf{s}(t)) - p_i(\mathbf{x}^i(t))]$, with $p_i(\mathbf{x}^i(t)) = \beta_i H(\mathbf{x}^i(t))$. Now, we can find a set of N_D driver nodes using steps 1 – 4 of our scheme to steer the controlled system to its synchronized state for the Erdős-Rényi (ER) and scale-free (SF) networks investigated here (cf. Figs. S1 and S3). The stability – under the terminology of the MSF – can be defined for the cases that satisfy $\sigma\lambda_2 > 0.36$ with λ_2 being the smallest non-zero eigenvalue of the control effective matrix $\mathbf{M}$ or equivalently, the maximum of the associated Jacobian evaluated at the fixed point to be less than or equal to -0.2.

Steps 1 – 4 can be either applied directly to the Jacobian (evaluated at the fixed point) of the system, immediately after block-diagonalizing it in the eigen-space of the Laplacian (*2*) or one can iteratively pin those nodes for which λ_2 of $\mathbf{M}$ increases the most until the critical condition just mentioned is met.

We have chosen three different combinations of the coupling strength σ and the pinning strength β (note that here β denotes the coefficient in the pinning function $p_i(\mathbf{x}^i(t)) = \beta_i H(\mathbf{x}^i(t))$

as $\{(\sigma, \beta)\} \in \{((2,8); (8,2); (8,8)\}$, from a rather small to a comparatively large value and carried out simulations for each combination. We find for ER networks the pinning density $n_D = N_D/N$ to decrease with increasing N, depending on the wiring probability p and the values of σ and β, however, an increasing trend is present at larger N (see Fig. S1).

We also observe that the fraction of required driver nodes decreases to a saturation level with increasing p for a fixed network size of $N = 120$. For SF networks, we find a monotonically increasing behavior of n_D with increasing network size for all three different combinations of σ and β.

Fig. S2 demonstrates how MSF-derived driver nodes distinguish themselves by specific properties. We find that in the case of ER networks the driver nodes are chosen from comparatively larger-degree nodes ($\langle k_D \rangle \simeq 12$ while $\langle k \rangle \simeq 10$), however, the distribution of natural frequencies does not differ that much from the overall distribution. We also find that in the case of SF networks the natural frequencies of the driver nodes demonstrate a comparatively different distribution from the overall distribution, not as distinguished as what we observed before in Fig. 3 (main text). The degree distribution is also comparable to the overall distribution.

In Fig. S3, we show how pinning density depends on pinning strength β and on global coupling strength σ. For large values of β and σ, we find $N_D \to 2$ for ER networks however, $N_D \to 20$ for SF coupling topology, for both out of $N = 120$ nodes. For small values of β and σ, we find $N_D \simeq N$, which means that all nodes should be controlled.

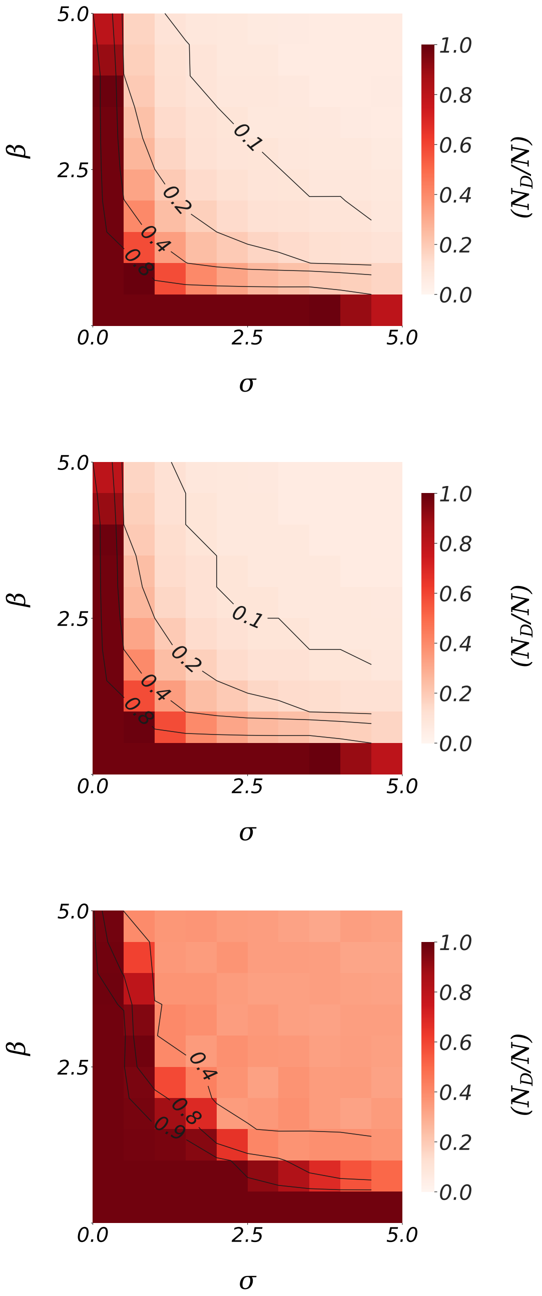

Figure S3: Pinning density $n_D = N_D/N$ for ER (top: $p = 0.3$; middle: $p = 0.8$) and SF networks (bottom) of size $N = 50$ for second-order Kuramoto oscillators depending on pinning strength β and global coupling strength σ in MSF-based control scheme. Results are averaged over 100 realizations at each point.

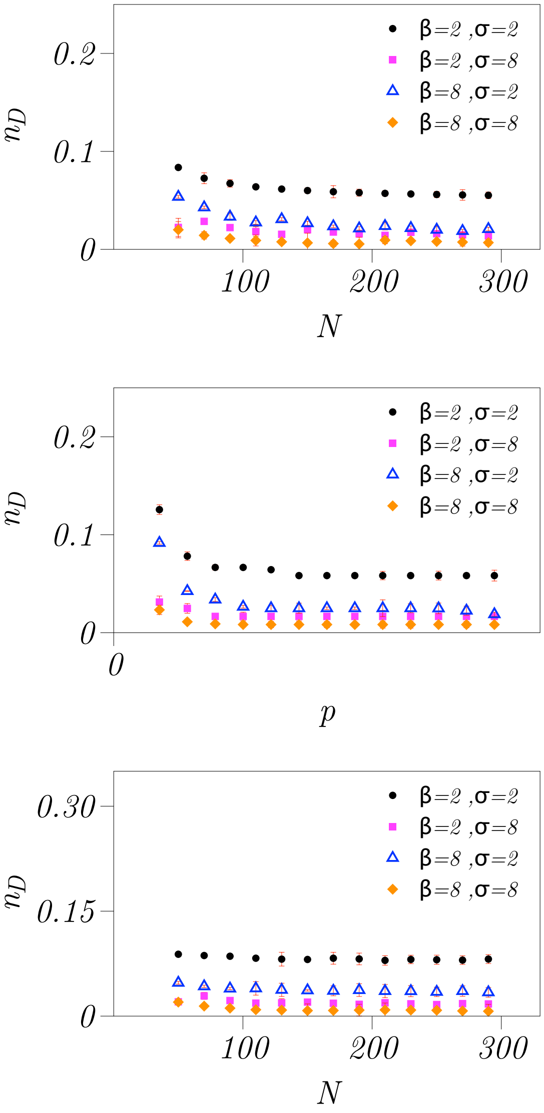

Figure S4: Pinning density n_D (fraction of driver nodes) derived with the MSF-based control scheme versus N and p for all combinations of $\{\sigma, \beta\} = \{2, 2; 2, 8; 8, 2; 8, 8\}$. Top: Erdős-Rényi networks (wiring probability $p = 0.3$), middle: Erdős-Rényi networks with different wiring probabilities for $N = 120$, bottom: scale-free networks of Rössler oscillators. Results are averaged over 100 realizations at each point. See Figure 5 in main text.

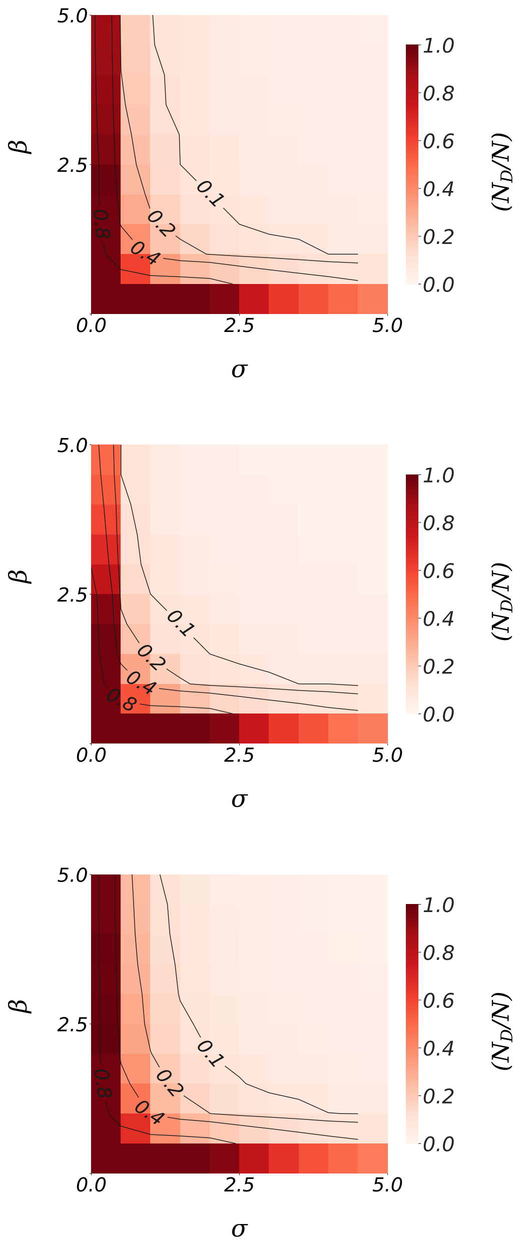

Figure S5: Pinning density $n_D = N_D/N$ for ER (top: $p = 0.3$; middle: $p = 0.8$) and SF networks (bottom) of size $N = 50$ for Rössler oscillators in the oscillatory regime depending on pinning strength β and global coupling strength σ with the MSF-based control scheme. Results are averaged over 100 realizations at each point.

Identifying driver nodes for networks of Rössler oscillators with the master stability function formalism

We consider a network of N Rössler oscillators, whose general dynamics is given by equation (5) (main text) with the 3-dimensional internal state

$$\mathbf{x}^i = \begin{bmatrix} x_1^i \\ x_2^i \\ x_3^i \end{bmatrix}, \; F(\mathbf{x}^i) = \begin{bmatrix} -x_2^i - x_3^i \\ x_1^i + a x_2^i \\ b + (x_1^i - c)x_3^i \end{bmatrix}, \; H(\mathbf{x}^i) = \begin{bmatrix} x_1^i \\ 0 \\ x_3^i \end{bmatrix}.$$

The function H couples oscillators through their x_1 and x_3 components. By linearizing equation (S.1) around the desired state $\mathrm{s}(t)$ and applying the control scheme using the master stability function (MSF) formalism, we can apply steps 1 – 4 of our proposed method to find a finite set of driver nodes. First, we consider Rössler oscillators with an *oscillatory* dynamics ($a = -0.2$, $b = 0.2$, $c = 5.7$) coupled through Erdős-Rényi (ER) networks and scale-free (SF) networks. Fig. S4 shows the dependence of the required pinning fraction $n_D = N_D/N$ for a stable oscillatory desired state versus network size for ER and SF networks and also versus the wiring probability for fixed size ($N = 120$) ER networks. We find that for both ER and SF networks, n_D decreases with an increasing size and in the case of fixed-size ER networks, n_D decreases to a final saturation level with increasing wiring probability p. Also, in Fig. S5, we show how pinning density n_D depends on pinning strength β and on global coupling strength σ.

We have carried out similar analyses for Rössler oscillators with chaotic dynamics ($a = 0.2$, $b = 0.2$, $c = 7$) with the same dynamical coupling function. The method presented for finding the driver nodes is not applicable here, because the final desired state will be a chaotic attractor and not just a fixed point. Thus, we will use an alternative algorithm which is of similar iterative nature.

We know that using the MSF-based control formalism, we can reduce the controllability or the stability problem to a simple inequality relation in which the ratio of the largest eigenvalue λ_{N+1} of the effective coupling matrix M over the corresponding smallest non-zero eigenvalue

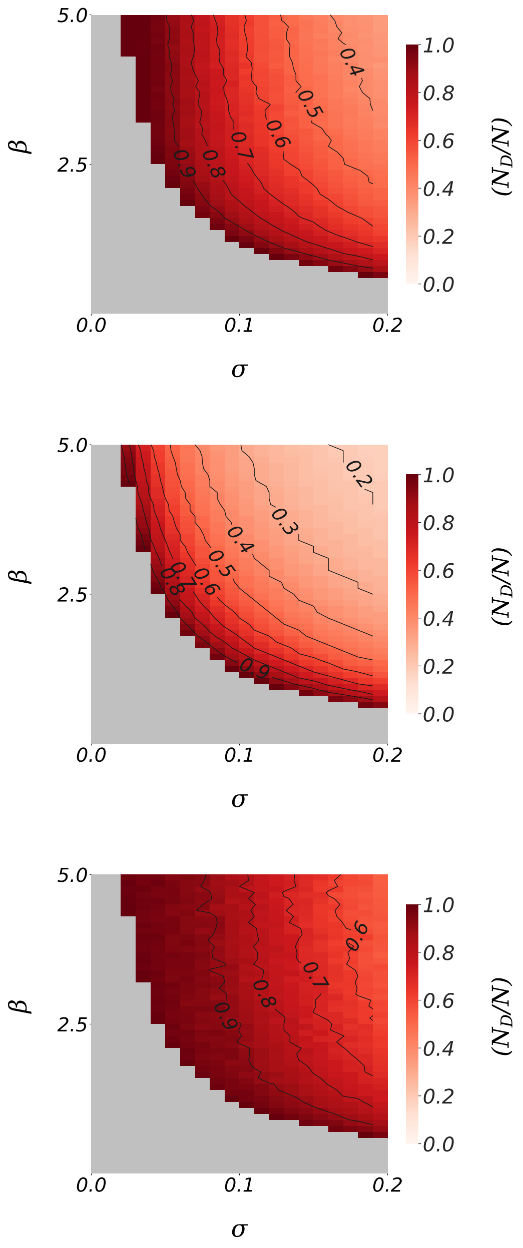

Figure S6: Pinning density $n_D = N_D/N$ for ER (top: $p = 0.3$; middle: $p = 0.8$) and SF networks (bottom) of Rössler oscillators in the chaotic regime depending on pinning strength β and global coupling strength σ. Mean values of n_D from ten realizations of each network with resolution $\Delta\sigma = 0.01$ and $\Delta\beta = 0.1$. The gray-shaded area marks a parameter range for which it is impossible to assure linear stability of the synchronized state by controlling the network.

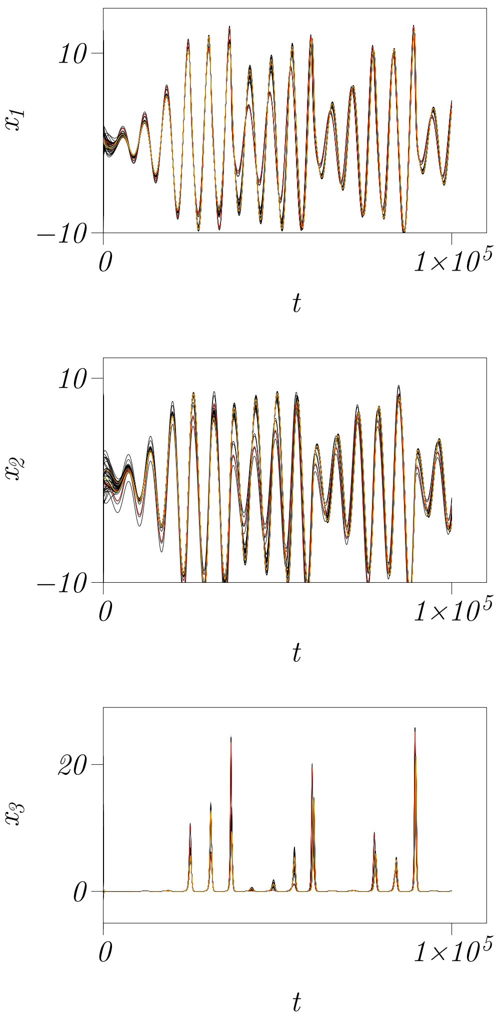

Figure S7: Exemplary temporal evolutions of components x_1, x_2, and x_3 of $N = 50$ chaotic Rössler oscillators controlled by $N_D = 6$ driver nodes with pinning strength $\beta = 3.2$ the same for each driver node and global coupling strength $\sigma = 0.8$.

λ_2 must be smaller than some constant α_2/α_1, with α_1 and α_2 determining the bounds within which the largest Lyapunov exponent of the linearized system would be negative – for the system under study determined to be $(\alpha_1, \alpha_2) = (0.13, 4.86)$ – to assure the linear stability of the final synchronized state. Here, we will iteratively pin the nodes for which the ratio λ_{N+1}/λ_2 of M becomes the smallest possible, until the inequality relation is satisfied for the first time.

The dependence of pinning density $n_D = N_D/N$ on pinning strength β and on global coupling strength σ is summarized in Fig. S6. It is interesting to note that for both small and large values of β and σ there is no controllability of this system, and depending on the network topology there are some intervals for β and σ for which the system is controllable. Also, in Fig. S7 exemplary temporal evolutions of components x_1, x_2, and x_3 of $N = 50$ Rössler oscillators with chaotic dynamics controlled with $N_D = 6$ driver nodes are illustrated.

Second-order Kuramoto-like dynamics on a power grid

A coarse-scale model describing power grid dynamics is the second-order Kuramoto-like model, for which we will provide some details in this section.

Basic elements of a power grid are generators (representing the power plants) with a positive power input ($P_j^{\mathrm{s}} > 0$) and motors (representing the consumers) with a negative power intake ($P_j^{\mathrm{s}} < 0$). Generators convert other kinds of energy to electrical energy, while motors do the reverse. It should be noted that in some of the generators, the power input can be noisy due to the randomness in their renewable energy sources. The dynamical variables which determine the state of some node j in the power grid are $\phi_j(t) = \Omega t + \theta_j$ and its time derivative $\dot{\phi}_j(t)$, where Ω (which is either $2\pi\times$ 50 Hz or $2\pi\times$ 60 Hz, depending on the country) is the reference or power line frequency at which (or close to) all machines oscillate and θ_j is the deviation from this reference. According to the law of conservation of energy, the power P_j^{s} produced or

consumed by node j ($j \in N$, where N denotes the size of the power grid) must be equal to the sum of its dissipated power P_j^{d}, its accumulated power P_j^{a}, and the power exchanged with the grid P_j^{t} (*3*)

$$P_j^{\mathrm{s}} = P_j^{\mathrm{d}} + P_j^{\mathrm{a}} + P_j^{\mathrm{t}} = P_j^{\mathrm{d}} + P_j^{\mathrm{a}} + \sum_{k=1}^{N} P_{jk}^{\mathrm{t}}, \tag{S.13}$$

where P_{jk}^{t} denotes the power transmitted from node k to node j. The dissipated energy at a machine is proportional to the square of the angular velocity, i.e., $P_j^{\mathrm{d}} = K_D \dot{\phi}_j^2$, where K_D is the dissipation coefficient and, for convenience, is assumed to be equal for all nodes. The accumulated kinetic energy is $P_j^{\mathrm{a}} = \frac{d}{dt}(\frac{1}{2} I_j \dot{\phi}_j^2)$, where I_j is the moment of inertia of node j, and the transmitted power is given by $P_{jk}^{\mathrm{t}} = -P_{jk}^{\max} \sin(\theta_j - \theta_k)$, where $P_{jk}^{\max}$ is the capacity of the link connecting nodes j and k for power transmission. By substituting these values into equation (S.13), we get N coupled differential equations for ϕ_j

$$P_j^{\mathrm{s}} = K_D \dot{\phi}_j^2 + I_j \ddot{\phi}_j \dot{\phi}_j - \sum_{k=1}^{N} P_{jk}^{\max} \sin(\phi_j - \phi_k). \tag{S.14}$$

Further substituting $\phi_j = \Omega t + \theta_j$ in equation (S.14) and assuming that the change of the deviation is much slower than the reference's change (i.e., $|\dot{\theta}_j| \ll \Omega$) leads to the following equation of motion

$$I_j \Omega \ddot{\theta}_j = (P_j^{\mathrm{s}} - K_D \Omega^2) - 2 K_D \Omega \dot{\theta}_j + \sum_k P_{jk}^{\max} \sin(\theta_j - \theta_k), \tag{S.15}$$

which is similar to the dynamics of a second-order Kuramoto oscillator (see equation (1) in main text),

$$M_j \ddot{\theta}_j = \omega_j - \alpha \dot{\theta}_j + \sum_{k=1}^{N} \lambda_{jk} \sin(\theta - \theta_k), \tag{S.16}$$

where M_j is the inertia coefficient. This system has a stable fixed point with constant phases $\tilde{\theta} := (\tilde{\theta}_1, \tilde{\theta}_2, \dots, \tilde{\theta}_N)$ and vanishing frequency deviations $\tilde{\nu} := (0, 0, \dots, 0)$ if the power grid is operating stable and reaches perfect synchrony.

As discussed in the main text, generators with renewable energy sources have noisy power inputs P_j^{s}, and therefore, their natural frequencies ω_j exhibit some fluctuations. One can take into account these fluctuations by adding an extra noise term to equation (S.15) for the power input P_j^{s} or similarly to equation (S.16) for the natural frequency ω_j. However, since the natural frequency of a rotor is directly related the power input (see Table (S1)), fluctuations in natural frequencies will have the same statistics as fluctuations in the power input except for a shift in the mean value. In real power grids, noise due to renewable energies has long-time correlations with non-Gaussian incremental distributions (*4, 5*). For simplicity, here we consider it to have white noise characteristics, and to guarantee positivity of the power input we consider uniformly distributed noise.

In the following, we derive an expression for the coupling strength λ_{jk} in equation (S.16) that is based on the known parameters of buses and transmission lines of a power grid. The link which connects nodes j and k is characterized by its impedance $z_{jk} = r_{jk} + ix_{jk}$, where r_{jk} is the link's resistance and x_{jk} its reactance. The inverse of the impedance is called admittance $y_{jk} = z_{jk}^{-1}$, a complex variable that can be written as $y_{jk} = g_{jk} + ib_{jk}$ with the conductance g_{jk} and the susceptance b_{jk}.

We define the nodal admittance matrix $\mathbf{Y}$ as

$$Y_{jk} = \begin{cases} \sum_l A_{jl} y_{jl} & \text{if } j = k \\ -y_{jk} & \text{if } j \neq k \text{ and } A_{ij} = 1 \\ 0 & \text{otherwise} \end{cases} \tag{S.17}$$

where A_{jk} denotes a component of the adjacency matrix $\mathbf{A}$. In other words, the admittance matrix is a generalization of the Laplacian matrix with admittances y_{jk} as weights. The net

current I_j passing through node j is given by (*6*)

$$\begin{aligned} I_j &= \sum_k A_{jk} y_{jk} (V_j - V_k) = V_j \sum_k A_{jk} y_{jk} - \sum_k y_{jk} V_k \\ &= V_j Y_{jj} - \sum_k A_{jk} y_{jk} V_k = \sum_k (Y_{jk}\delta_{jk} - A_{jk} y_{jk}) V_k \\ &= \sum_k Y_{jk} V_k, \end{aligned} \tag{S.18}$$

which is Kirchhoff's law. The power is given by

$$\omega_j = \Re(V_j I_j^*) \tag{S.19}$$

where $\Re(z)$ is the real part of the complex variable z. By substituting I_j from equation (S.18) into equation (S.19) and writing voltages in their polar form (i.e., $V_j = |V_j| e^{i\theta_j}$) we get

$$\omega_j = \Re(|V_j| \sum_k Y_{jk} |V_k| e^{i(\theta_j - \theta_k)}) \tag{S.20}$$

For high-voltage transmission lines, resistance is negligible $(r_{jk} \approx 0)$ and admittance can be written as $y_{jk} = -\frac{i}{x_{jk}}$. This assumption simplifies equation (S.20) to

$$\omega_j = \sum_{k=1}^{N} A_{jk} \frac{|V_j||V_k|}{x_{jk}} \sin(\theta_j - \theta_k), \tag{S.21}$$

which is apparently the steady state of equation (S.16) and is satisfied if the system has reached perfect synchrony. From this analogy, we can argue that for any link between nodes i and j in a power grid, the coupling strength λ_{ij} equals the product of the absolute values of the nodes' voltages divided by the reactance of the transmission line (i.e., $\lambda_{ij} = \frac{|V_i||V_j|}{x_{ij}}$).

In Tab. (S1), we report the relationship between control parameters of second-order Kuramoto oscillators (see equation (S.16)) and the topological and dynamical control parameters of a power grid. To find the motion of all phase rotors in a power grid, one can numerically integrate equation (S.16) with specific initial conditions.

Table S1: Relationship between control parameters of second-order Kuramoto oscillators and control parameters of a power grid

Kuramoto oscillator	power grid
M_j	$I_j \Omega$
ω_j	$P_j^{\mathrm{s}} - K_D \Omega^2$
α	$2 K_D \Omega$
λ_{jk}	$A_{jk} \frac{\lvert V_j \rvert \lvert V_k \rvert}{x_{jk}}$

References

1. F. Sorrentino, M. di Bernardo, F. Garofalo, G. Chen, Controllability of complex networks via pinning. *Phys. Rev. E* **75**, 046103 (2007).

2. L. M. Pecora, T. L. Carroll, Master stability functions for synchronized coupled systems. *Phys. Rev. Lett.* **80**, 2109–2112 (1998).

3. G. Filatrella, A. H. Nielsen, N. F. Pedersen, Analysis of a power grid using a Kuramoto-like model. *Eur. Phys. J. B* **61**, 485–491 (2008).

4. P. Milan, M. Wächter, J. Peinke, Turbulent character of wind energy. *Phys. Rev. Lett.* **110**, 138701 (2013).

5. M. R. R. Tabar, M. Anvari, G. Lohmann, D. Heinemann, M. Wächter, P. Milan, E. Lorenz, J. Peinke, Kolmogorov spectrum of renewable wind and solar power fluctuations. *Eur. Phys. J. Spec. Top.* **223**, 2637–2644 (2014).

6. A. Plietzsch, P. Schultz, J. Heitzig, J. Kurths, Local vs. global redundancy–trade-offs between resilience against cascading failures and frequency stability. *Eur. Phys. J. ST* **225**, 551–568 (2016).
